# Safety and efficacy of colistin and fluoroquinolone in neonatal persistent late-onset sepsis

**DOI:** 10.1016/j.jsps.2021.07.014

**Published:** 2021-07-21

**Authors:** Mountasser Al-Mouqdad, Khalid Eljaaly, Ayman Abdalgader, Maha Al-Anazi, Muhammed Taha, Arwa Alshaibani, Raneem Asfour, Thanaa Khalil, Suzan Asfour

**Affiliations:** a Neonatal Intensive Care Unit, Hospital of Paediatrics, King Saud Medical City, Riyadh, Saudi Arabia; b Faculty of Pharmacy, King Abdulaziz University, Jeddah, Saudi Arabia; c Pharmacy Department, Pharmaceutical Care Services, King Saud Medical City, Riyadh, Saudi Arabia; d General Paediatrics Department, Hospital of Paediatrics, King Saud Medical City, Riyadh, Saudi Arabia; e Pharmacy College, Jordan University of Science and Technology, Irbid, Jordan; f Obstetrics and Gynecology Department, Maternity Hospital, King Saud Medical City, Riyadh, Saudi Arabia; g Clinical Pharmacy Department, Pharmaceutical Care Services, King Saud Medical City, Riyadh, Saudi Arabia

**Keywords:** Multi-drug resistant organisms, Antibiotics, Colistin, Fluoroquinolone, Premature infants, Late-onset sepsis

## Abstract

Growing resistance of microorganisms to antibiotics for the treatment of late-onset sepsis (LOS) in premature infants has led physicians to use antibiotics that are not well studied in neonatal populations. We aimed to determine the efficacy and safety of colistin and fluoroquinolone for the treatment of persistent LOS. We retrospectively reviewed infants with gram-negative LOS, who received either colistin or fluoroquinolone therapy, to determine if there was a significant difference in kidney and liver function tests and electrolyte levels before, during, and at the end of the treatment. Infants who received colistin and fluoroquinolone had 17 and 34 positive cultures with gram-negative organisms, respectively. Multi-drug resistant organisms were more common in infants who received colistin than in those who received fluoroquinolone. Microbiological clearance was found to be higher in infants treated with fluoroquinolone than in those treated with colistin. In both the groups, the median levels of kidney and liver function tests and electrolytes showed a significant increase during the treatment. The prescription of colistin and fluoroquinolones should be reserved for cases with no other safe and effective alternatives.

## 1 Introduction

Neonatal sepsis is one of the main causes of neonatal morbidity and mortality, especially among premature infants (Stoll et al., 2002, Shane and Stoll, 2014, Liu et al., 2016). The duration and type of antibiotics for management of neonatal sepsis in premature infants remain a controversial matter in neonatal practice: Premature infants have weak innate immunity, they stay longer in the neonatal intensive care unit (NICU) and are more exposed to invasive and noninvasive procedures, and finally, they manifest sepsis with nonspecific symptoms (Callaghan et al., 2006, Beck et al., 2010). Therefore, the treatment of neonatal sepsis, especially that acquired after birth and during hospitalization, is a challenge to neonatologists.

Late-onset sepsis (LOS) is caused by various microorganisms. In our previous study addressing the responsible organisms and antibiotic susceptibility, we isolated 70 organisms responsible for LOS. Gram-positive organisms were most common, but gram-negative (GN) organisms were most lethal (Al-Mouqdad et al., 2018).

Although several modalities have been developed for the early detection and treatment of LOS, they have failed to reduce the incidence of LOS (Griffin et al., 2007, Sullivan and Fairchild, 2015, Mithal et al., 2018). On the contrary, the arbitrary use of antibiotics has increased, even with a low probability of sepsis, and the subsequent proportion of neonatal morbidities and antibiotic resistance have become more prominent (Asfour and Al-Mouqdad, 2019, Lessa et al., 2009, Cantey and Milstone, 2015).

Consequently, escalating bacterial resistance has led to the creation of multi-drug resistant organisms (MDROs). In a recent study, GN MDROs caused approximately 20% of the episodes of GN sepsis (Tsai et al., 2014), which led to the reintroduction of antibiotics without strong safety records. Colistin and fluoroquinolones are two such antibiotics that are frequently used without clear evidence regarding their efficacy and safety (Jajoo et al., 2011, Alan et al., 2014, Al-Lawama et al., 2016, Newby et al., 2017).

Therefore, our objectives in the present study were as follows: (1) to retrospectively review the medical records of our patients who received either colistin or fluoroquinolone to evaluate their efficacy and safe use and (2) to determine recommendations that should be provided to neonatologists and infectious disease physicians for the management of LOS with GN organisms.

## 2 Material and methods

### 2.1 Study design

This study was a retrospective cohort review of the charts of infants with culture-proven GN organisms admitted to the NICU of our institution from January 2015 to September 2020.

The NICU has an average annual admission of 1100 patients, including NICU levels 2 and 3. This study was conducted in accordance with the Declaration of Helsinki and Good Pharmacoepidemiology Practices guidelines and was approved by the medical ethical review committee of our institution with a waiver of consent (reference number H1RI-25-Feb19-01).

### 2.2 Inclusion and exclusion criteria

Infants included in this study were those admitted at KSMC with GN bacteria who received either colistin or fluoroquinolone (ciprofloxacin or levofloxacin) for 48 h or longer. Patients were excluded if they had received previous therapy for less than 48 h or had major congenital anomalies.

### 2.3 Data collection and follow-up

We retrospectively reviewed all the included patients’ charts from the time of NICU admission until discharge or death. Demographic and clinical data, source of infection, laboratory tests including microbiological studies (blood, urine, CSF, antimicrobial sensitivity, culture results, and isolated pathogens), age at colistin or fluoroquinolone treatment initiation, duration of treatment, concomitant antibiotic treatments, and clinical outcomes data were obtained for all the infants. Maternal data, including the presence of premature rupture of membrane, antenatal steroid treatment, and mode of delivery, were also obtained. In addition, data from laboratory tests, such as renal (creatinine and urea levels) and liver (ALT, AST, and bilirubin levels) function tests, serum electrolytes (calcium, magnesium, potassium, sodium, and phosphorus) at the beginning of the treatment, during colistin and fluoroquinolone treatment, and at the end of the treatment were also collected to assess possible adverse effects.

The primary outcome of the study was microbiological clearance, which measured the incidence of clean cultures (blood, CSF, urine, and tracheal aspirate) after at least 3 days of starting colistin or fluoroquinolone.

The secondary outcome was the occurrence of adverse events during colistin and fluoroquinolone therapy.

### 2.4 Statistical analysis

Before starting the analysis, the dataset was checked for missing variables. Data were analyzed using Statistical Package for the Social Sciences version 25.0 (SPSS Inc., Chicago, IL, USA). Descriptive statistics, including means and standard deviations, medians, 25th and 75th percentiles, frequencies, and percentages, were used to describe the maternal and neonatal variables depending on distribution.

A Fisher’s exact test or Chi-squared test was used to determine the association between categorical variables, as appropriate. A Mann–Whitney *U* test was used for ordinal qualitative variables (gestational age, birth weight, and Apgar score). For continuous variables, an unpaired Student’s *t*-test was used; when data were not normally distributed, a Mann–Whitney *U* test was performed. A Kolmogorov–Smirnov test and visual inspection of histograms were performed to verify the normality of distribution of the quantitative variables. Friedman two-way analysis of variance by ranks was used to determine if there was a significant difference in kidney and liver functional tests and electrolytes levels between the first day, during, and at the end of the treatment, for both colistin and fluoroquinolone. Later, when the p-value from the Friedman test was statistically significant, a Wilcoxon signed-rank test was used to determine which time point differed from others. Kidney and liver functional tests and electrolyte values at the beginning of colistin and fluoroquinolone treatment were compared with the maximum value during the treatment, as well as with the value at the end of the treatment, using a non-parametric test (Wilcoxon signed-rank test). A Pearson test was used for correlation analysis between serum creatinine and potassium levels. Kaplan–Meier survival curves with log-rank tests were used to compare survival among those who received colistin versus those who received fluoroquinolone. All statistical tests were two-tailed, and p-values < 0.05 were considered statistically significant.

## 3 Results

Forty-two infants met the inclusion criteria and were eligible for the final analysis. There were no significant differences between the prenatal and postnatal demographic characteristics of the mothers and infants who were treated with either colistin or fluoroquinolone (Table 1).

**Table 1:** Demographic and clinical characteristics of mothers and infants with gram-negative infections treated with colistin or fluoroquinolone (n = 42).

**Parameter**	**Colistin (n = 15)**	**Quinolone (n = 27)**	**p-value**
Antenatal steroid treatment	4 (26.7)	10 (37)	0.495
Cesarean section delivery	9 (60)	15 (55.6)	0.780
Maternal hypertension	2 (13.3)	1 (2.4)	0.287
Gestational diabetes mellitus	0 (0)	1 (2.4)	1
PROM	0 (0)	1 (2.4)	1
Gestational age, weeks	27 (25–34)	28 (25–34)	0.853
Birth weight, grams	840 (740–1580)	1140 (775–1740)	0.529
1-min Apgar score	5 (2.75–6)	4 (3–6)	0.551
5-min Apgar score	7 (5.25–7.75)	7 (6–8)	0.720
Male sex	7 (46.7)	15 (55.6)	0.580
Inotropes	11 (73.3)	19 (70.4)	0.839
EOS	0 (0)	1 (3.7)	1
Survival	7 (36.7)	9 (33.3)	0.394
Age of starting colistin or quinolone	28 (16–92)	48 (21.25–67.75)	0.697
Duration of therapy	17 (8–28)	9 (6–15)	0.105
Total days of antibiotics	46 (38–94)	67 (52–91)	0.329
LOHS	99 (49–116)	96 (57–120)	0.753
Gram-positive infection	10 (66.7)	19 (70.4)	1
MDROs	8 (53.3)	5 (18.5)	0.035*
Microbiological clearance	7 (46.7)	23 (85.2)	0.013*

Types of catheter, n (%)			
UAC	5 (33.3)	13 (48.1)	0.353
UVC	9 (60)	19 (70.4)	0.495
PICC	7 (46.7)	13 (48.1)	0.927
CVC	5 (33.3)	10 (37)	0.810

Source of infection, n (%)			
Bacteremia	10 (66.7)	13 (48.1)	0.248
VAP	6 (40)	19 (70.4)	0.055
Meningitis	1 (6.7)	1 (3.7)	1

Concomitant antimicrobials and antifungal treatment during colistin or fluoroquinolone administration, n (%)			
Cotrimoxazole	2 (13.3)	7 (25.9)	0.451
Amphotericin b	2 (13.3)	5 (18.5)	1
Vancomycin	6 (40)	5 (18.5)	0.158
Meropenem	6 (40)	10 (37)	0.85
Caspofungin	1 (6.7)	2 (7.4)	1
Amikacin	0 (0)	5 (18.5)	0.142
Fluconazole	0 (0)	1 (3.7)	1
Linezolid	2 (13.3)	3 (11.1)	1
Cefepime	2 (13.3)	3 (11.1)	1
Daptomycin	1 (6.7)	3 (11.1)	1
Rifampicin	1 (6.7)	3 (14.8)	1
Clindamycin	1 (6.7)	0 (0)	0.357
Gentamicin	4 (26.7)	2 (7.4)	0.164
Ceftazidime	2 (13.3)	0 (0)	0.164
Cloxacillin	1 (6.7)	0 (0)	0.357
Metronidazole	1 (6.7)	1 (3.7)	1
Imipenem	1 (6.7)	1 (3.7)	1
Piperacillin–Tazobactam	0 (0)	1 (3.7)	1
Penicillin G	0 (0)	1 (3.7)	1

PROM, premature rupture of membrane; EOS, early-onset sepsis; LOHS, length of hospital stay; MDROs, multi-drug resistant organisms; UAC, umbilical arterial catheter; UVC, umbilical venous catheter; PICC, peripherally inserted central catheter; CVC, central venous catheter; VAP, ventilator associated pneumonia. Data are presented as the number (%) or median (IQR) as appropriate. *p-values < 0.05 are considered statistically significant.

There were differences in comorbidities, sites of infection, types of catheter, responsible pathogens, concomitant antibiotics, and survival rates between the groups; however, these differences did not reach statistical significance. All the infants received other antimicrobial agents prior to colistin or fluoroquinolone therapy. The most common concomitant antimicrobials were meropenem (38.1%) and vancomycin (26.2%).

There were 51 positive cultures in total. Infants who received colistin and fluoroquinolone had 17 and 34 positive cultures with GN organisms, respectively. Twenty-seven patients received fluoroquinolone with 34 positive cultures. Most patients presented with sepsis as VAP, with six infants producing positive cultures from more than one site. However, the organisms were primarily isolated from blood in the colistin group, with two infants who had positive cultures from more than one site.

**Fig. 1:**
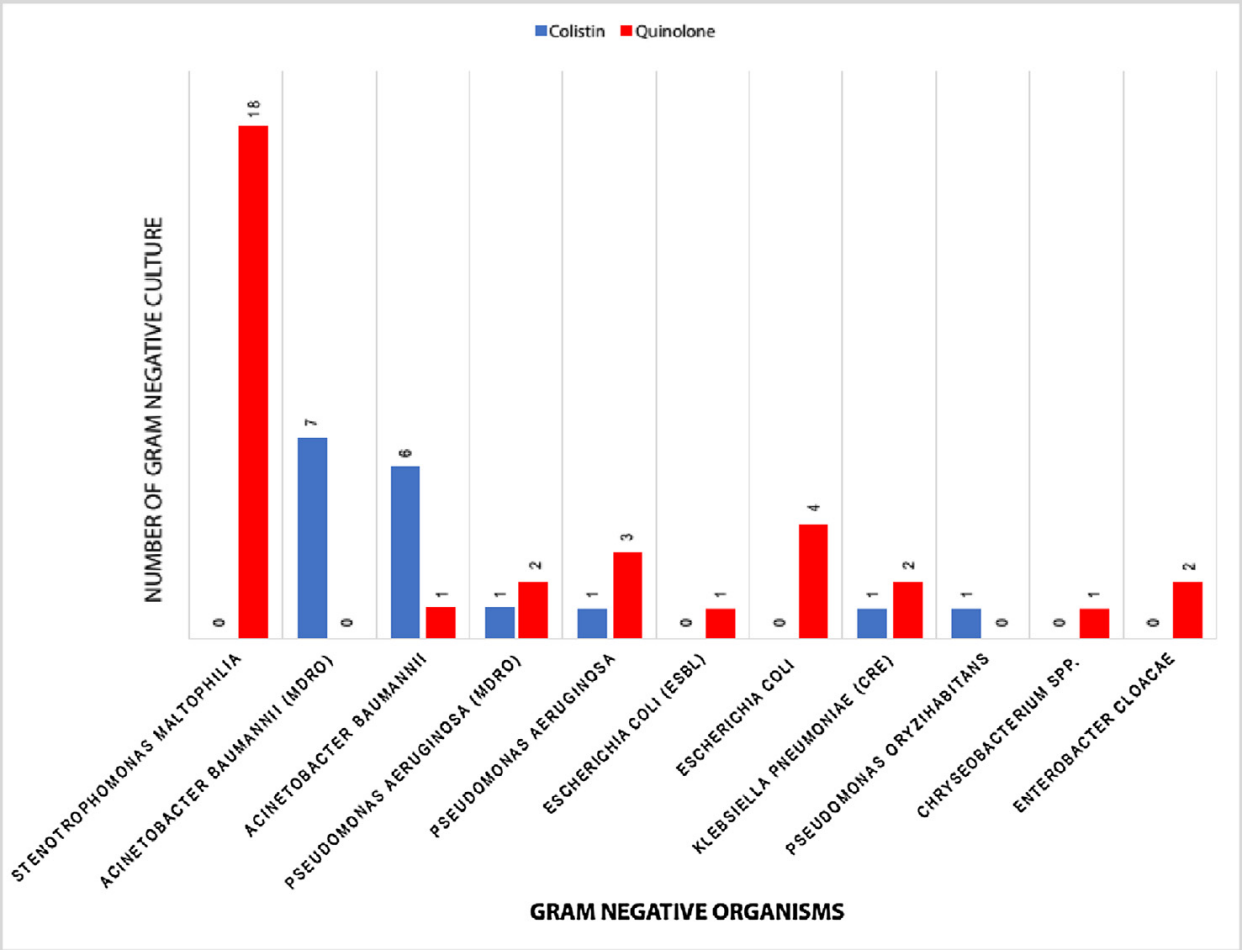
Types of gram-negative bacteria among infants who received either colistin or quinolone therapy.

Fig. 1 shows the distribution of GN organisms, including MDRO-GN *Acinetobacter baumannii*, *Pseudomonas aeruginosa*, *Escherichia coli* Extended Spectrum Beta-Lactamase (ESBL), and carbapenem-resistant *Enterobacteriaceae*, according to colistin and fluoroquinolone therapy. *A. baumannii*, including multi-drug resistant *Acinetobacter*, was the most common causative pathogen in infants treated with colistin (76.5%). *Stenotrophomonas maltophilia* was the most isolated organism in the fluoroquinolone group cultures (53%), followed by *E. coli* (14.7%), including *E. coli* ESBL (Fig. 1). One infant in the quinolone group had two isolated organisms from one blood culture (*A. baumannii* and *E. coli* ESBL). There were nine (53%) MDROs isolated from infants treated with colistin, and five isolated from infants treated with quinolone (14.7%) (Fig. 1).

**Fig. 2:**
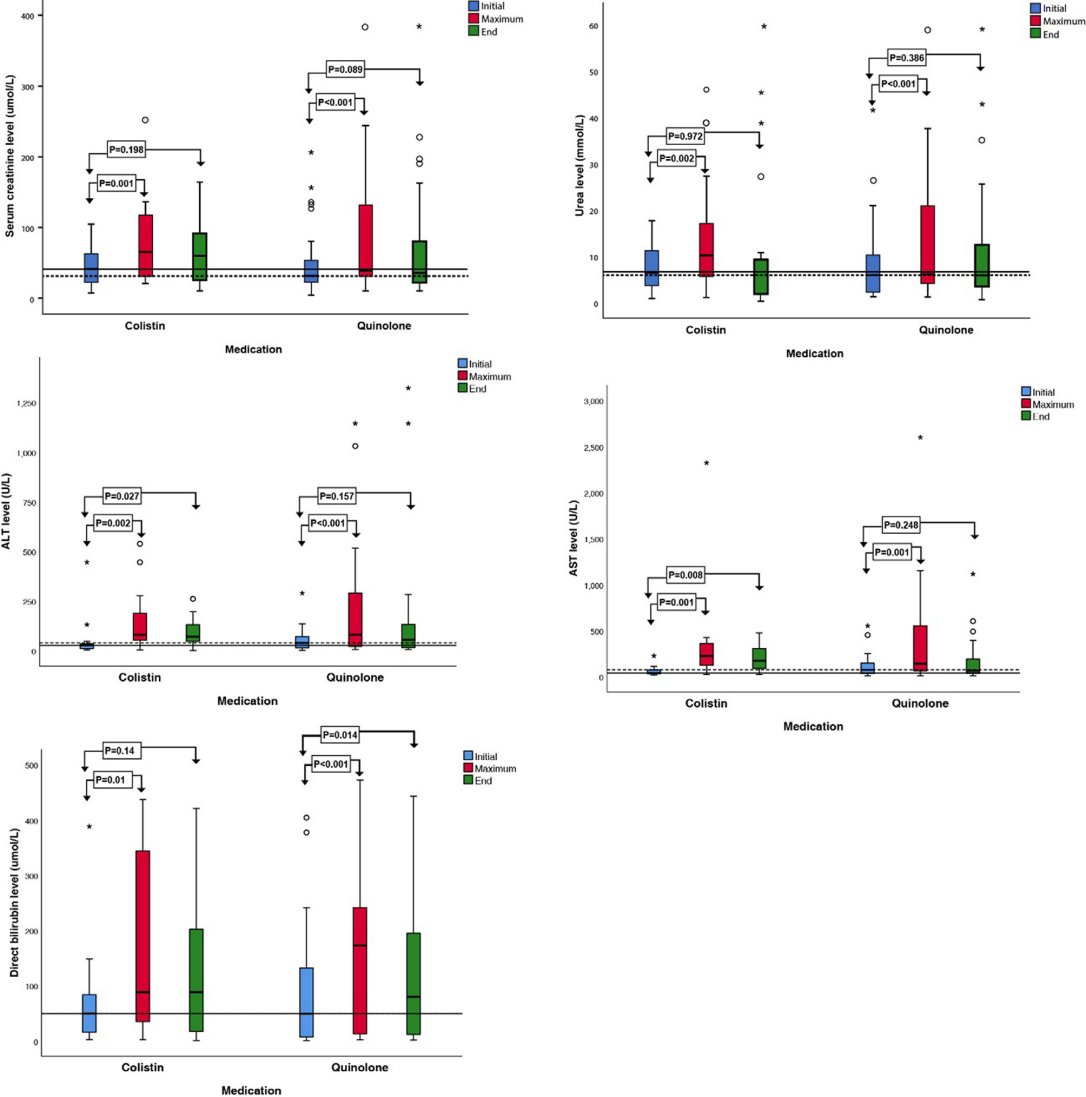
Boxplot of the distribution of serum creatinine, urea, ALT, AST, and direct bilirubin levels on the first day of treatment (Initial), at the peak value (Maximum), and at the end of treatment (End). The horizontal line within the boxes represents the median baseline value on the first day of colistin treatment. The dotted line within the boxes represents the median baseline value on the first day of fluoroquinolone treatment. ALT, alanine transaminase; AST, aspartate transaminase.

In both the groups, the median levels of creatinine, urea, ALT, AST, and direct bilirubin showed a significant increase during treatment (Table 2). The change between the levels at the beginning of the treatment and those at the end of the treatment was insignificant for creatinine and urea in both the groups; however, the change between the beginning and end of the treatment was significant for ALT and AST in the colistin group (P = 0.027 and P = 0.008, respectively) and for direct bilirubin in the fluoroquinolone group (P = 0.014) (Fig. 2). However, no significant differences in maximum values of these laboratory parameters were observed between those who received colistin and fluoroquinolones.

**Table 2:** Differences in kidney and liver function tests among the initial, maximum, and end values during the treatment of infants with colistin or fluoroquinolone medication.

	Colistin				Quinolone				Comparison
	Initial value	Maximum value	End value	p-value for change between therapy beginning, maximum and end	Initial value	Maximum value	End value	p-value for change between therapy beginning, maximum and end	p-value for difference between groups in maximum values
Creatinine (mmol/ml)	40.8 (18–62)	65 (30–132)	30.6 (24–102.5)	<0.001*	31.5 (20.95–9.98)	38.55 (30.03–134.83)	34 (20.63–95.6)	<0.001*	0.957
Urea	6.8 (2.69–12.9)	9.9 (5.5–17.6)	5.1 (2.1–10.9)	<0.001*	6.05 (2.45–12.43)	10.59 (4.3–21.5)	6 (3.6–12.73)	<0.001*	0.797
ALT (u/ml)	26.6 (10.1–39)	79.7 (50–196)	71 (44–148)	<0.001*	39 (13.13–71)	80 (20.92–292.37)	55.55 (14–136.63)	<0.001*	0.745
AST (u/ml)	37.8 (29–78)	225 (99.2–371)	172 (80–350)	<0.001*	73.15 (33–150.98)	139.4 (58.43–641.8)	64.3 (35.75–195.75)	<0.001*	0.646
Direct bilirubin	49.6 (13–90.8)	88.3 (20.1–388.2)	88.3 (15.1–264.6)	0.002*	49.4 (6.25–143.4)	172.7 (12.1–244.05)	79.8 (11.6–210.05)	<0.001*	0.555

ALT, alanine transaminase; AST, aspartate transaminase. Data are presented as the median (Interquartile range [IQR]). * p-values < 0.05 are considered statistically significant.

**Fig. 3:**
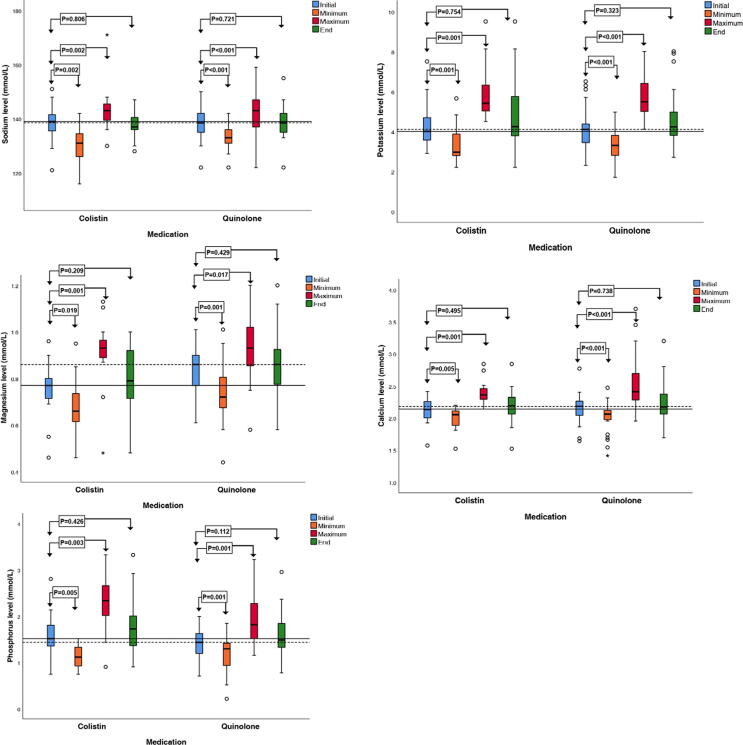
Boxplot of the distribution of serum sodium, potassium, magnesium, calcium, and phosphorus levels on the first day of treatment (Initial), at the peak value (Maximum), and at the end of treatment (End). The horizontal line within the boxes represents the median baseline value on the first day of colistin treatment. The dotted line within the boxes represents the median baseline value on the first day of fluoroquinolone treatment.

With respect to serum electrolyte levels, both colistin and fluoroquinolone treatment were associated with significant disturbances in sodium, potassium, calcium, magnesium, and phosphorus levels between the median values on the first day of treatment, and the median of the minimum and maximum values during treatment (Table 3). Like the renal function tests, there was no significant change between the beginning and end levels of electrolytes in both the groups (Fig. 3).

**Table 3:** Median (IQR) of the difference in electrolyte levels among initial, minimum, maximum, and end values during the treatment of infants with colistin or fluoroquinolone.

	Colistin					Quinolone				
	Initial value	Maximum value	Minimum value	End value	p-value	Initial value	Maximum value	Minimum value	End value	p-value
Sodium	139 (135–142)	143 (139–146)	131 (124 –135)	137 (136–141)	<0.001*	138.5 (134.75–142.5)	143 (136.5–147)	133 (131–136.25)	138.5 (134.75–142.5)	<0.001*
Potassium	4 (3.3–4.78)	5.31 (5.02–6.64)	2.96 (2.78 –4.16)	4.24 (3.57–6)	<0.001*	4.1 (3.42–4.71)	5.48 (4.9– 6.55)	3.3 (2.75–3.8)	4.23 (3.8–5.24)	<0.001*
Magnesium	0.77 (0.71–0.81)	0.93 (0.89–0.97)	0.66 (0.6 –0.76)	0.79 (0.71–0.92)	<0.001*	0.86 (0.76–0.9)	0.93 (0.85–1.02)	0.72 (0.66–0.82)	0.86 (0.77–0.94)	<0.001*
Calcium	2.14 (2.05–2.29)	2.36 (2.29–2.48)	2.05 (1.85–2.11)	2.19 (2.05–2.33)	<0.001*	2.18 (2.09–2.32)	2.41 (2.27–2.73)	2.06 (1.96–2.13)	2.17 (2.06–2.38)	<0.001*
Phosphorus	1.51 (1.27–1.86)	2.33 (1.96– 2.8)	1.11 (0.9– 1.38)	1.72 (1.34–2.06)	<0.001*	1.43 (1.19–1.62)	1.81 (1.47–2.34)	1.29 (0.89–1.42)	1.48 (1.32–1.9)	<0.001*

IQR, interquartile range. Data are presented as the median (IQR). * p-values < 0.05 are considered statistically significant.

We found no difference between courses of treatment in terms of how fast the liver function tests (LFTs), renal function tests (RFTs), and electrolytes reached peak levels after initiating treatment, except for AST and sodium levels. AST and sodium levels peaked three times faster in patients treated with fluoroquinolone (P = 0.032) than in those treated with colistin (P = 0.041) (Table 4).

**Table 4:** The day of treatment when maximum levels of RFT, LFT, and electrolytes were attained among infants treated with colistin or fluoroquinolone.

Parameter	Median (days) of colistin	Median (days) of quinolone	p-value
Creatinine	11 (4–12)	6.5 (1–11.25)	0.391
Urea	5 (2–12)	5.5 (1–9.5)	0.683
ALT	15 (4–20)	5 (2.75–11)	0.109
AST	15 (5–20)	5 (2.75–10.25)	0.032*
Direct bilirubin	8 (2–15)	6 (2–12)	0.765
Sodium	9 (3–19)	3.5 (1.75–8.25)	0.041*
Potassium	6 (4–12)	6 (4–12)	0.855
Magnesium	11 (6–15)	7 (5–11)	0.427
Calcium	5 (4–16)	7 (2–12)	1
Phosphorus	6 (3–11)	7 (3–10)	0.705

RFT, renal function test; LFT, liver function test; ALT, alanine transaminase; AST, aspartate transaminase. Data are presented as the median (IQR). * p-values < 0.05 are considered statistically significant.

There was a significant correlation between maximum serum creatinine and potassium levels (P = 0.003, r^2^ = 0.216). Furthermore, Kaplan–Meier curves demonstrated no relationship between either colistin or fluoroquinolone and survival rate (log-rank test, P = 0.539).

## 4 Discussion

We found that both colistin and fluoroquinolone were used to treat LOS caused by GN organisms, including MDROs. We demonstrated that colistin and fluoroquinolone were associated with impairments in hepatic and renal functions during treatment. Additionally, either an increase or decrease in serum electrolytes was found in both the treatment groups. Fluoroquinolones was associated with increases in AST and serum sodium levels faster than colistin.

Colistin was the primary choice to treat GN sepsis six decades ago; however, it was replaced with aminoglycosides due to nephrotoxicity and neurotoxicity (Falagas and Kasiakou, 2005, Li et al., 2006, Eljaaly et al., 2021, Owen et al., 2007, Poudyal et al., 2008). This led to the restriction of their use to only MDROs such as *A. baumannii* and *K. pneumoniae* (Falagas and Kasiakou, 2005, Li et al., 2006). In our study, we found that colistin was associated with impaired hepatic and renal functions and serum electrolytes during treatment; this disturbance also persisted for AST and ALT. Other studies pertaining to the use of colistin in term and preterm infants demonstrated a variety of renal impairments and electrolytes disturbances (Iosifidis et al., 2010, Jajoo et al., 2011, Alan et al., 2014, Jasani et al., 2016, Çağan et al., 2017, Ilhan et al., 2018). These studies showed heterogeneous results in terms of acute kidney injury: 19% in Alan et al. (Alan et al., 2014) and 0% in Jasani et al. (Jasani et al., 2016). Similarly, for serum electrolytes, Ipek et al. (Ipek et al., 2017) and Ilhan et al. (Ilhan et al., 2018) demonstrated hypokalemia and hypomagnesemia in patients receiving colistin; however, Alan et al. showed hypomagnesemia only (Alan et al., 2014), and Jasani et al. showed no electrolyte disturbance (Jasani et al., 2016).

Fluoroquinolones are broad-spectrum bactericidal antibiotics that have re-emerged after antibiotic resistance and have become more prominent (Jackson and Schutze, 2016). Despite precautions about their potential joint and musculoskeletal toxicities in adolescents, as well as the lack of specific information in neonates, they have been used to treat life-threatening infections in neonates (Forsythe and Ernst, 2007, Bradley and Jackson, 2011). Indications for fluoroquinolone treatment in our study were similar to those of colistin; they are prescribed for MDRO-GN and are associated with similar side effects. They were associated with renal and hepatic impairment and serum electrolyte disturbances during treatment. Direct bilirubin was more prominent than other multiorgan measurements and remained abnormal even after the fluoroquinolone course ended. A recent case series reported six cases of preterm and term infants treated with ciprofloxacin for MDRO-GN (Newby et al., 2017). Newby et al. stated that the outcome of treatment was successful without major renal, hepatic, or serum electrolyte disturbances (Newby et al., 2017). A systematic review on ciprofloxacin use in neonates was published nearly 10 years ago; Kaguelidou et al. collected five cohort studies and 27 single case reports; however, measurements of efficacy were only available in one cohort study (64%) (Kaguelidou et al., 2011). The other cohort studies only included infant survival; therefore, an estimation of clinical response was not possible.

The microbiological clearance in the present study was higher in the fluoroquinolone group (85%) than in the colistin group (53%). This may be attributed to the higher proportion of MDROs in the colistin group. By reviewing the rate of microbiological clearance on colistin efficacy in previous studies, we found that it ranged between 69% and 88% (Alan et al., 2014, Çağan et al., 2017, Ilhan et al., 2018). We subsequently delved into the reasons for this difference between our study and the others. We found that some studies included term infants in whom the response to treatment was greater than that of the premature infants (Jajoo et al., 2011; Lexicomp Lexicomp Online, 2020, Nakwan et al., 2019). In other studies, some patients received colistin empirically without waiting for culture results, which could be negative (Kaguelidou et al., 2011, Alan et al., 2014). Furthermore, the percentage of MDROs varied in some studies, despite not being mentioned (Jajoo et al., 2011, Alan et al., 2014).

There were some limitations of our study: (1) it was a retrospective study without a control group, (2) it was difficult to assess the neurologic complications in the colistin group as the patients were premature and markedly ill, and (3) the concomitant use of antibiotics may play a role in developing the systemic side effects in both groups; therefore, a well-designed prospective study is required to determine the efficacy and safety of colistin and fluoroquinolone in premature infants.

## 5 Conclusion

Our findings demonstrate that both colistin and fluoroquinolone used in the treatment of persistent or MDRO gram-negative LOS, associated with hepatic and renal function impairment and serum electrolyte disturbance; therefore, these medications must be used with caution, under particular circumstances, and when there is no safe and effective alternative. This study demonstrates: (1) the difficulty with regards to LOS treatment and the management of patients, especially those with MDRO-GN, (2) that colistin and fluoroquinolone have systemic side effects which require observation during treatment, and (3) that these patients require follow-up, even after discharge, to ensure that there are no long-term sequelae.

## Funding

This research did not receive any specific grant from funding agencies in the public, commercial, or not-for-profit sectors.

## Declaration of Competing Interest

The authors declare that they have no known competing financial interests or personal relationships that could have appeared to influence the work reported in this paper.
