# Supplementary material for: Safety and efficacy of colistin and fluoroquinolone in neonatal persistent late-onset sepsis
Source: Saudi Pharm J. 2021 Jul 21;29(9):1013–20. doi: 10.1016/j.jsps.2021.07.014 (PMC8463448; doi:10.1016/j.jsps.2021.07.014)
Supplement: Supplementary data 1 [file mmc1.docx]

*Definitions*

**EOS:** Sepsis caused by pathogens transmitted vertically from mother to infant, occurring in the first 3 days of [life](https://www.sciencedirect.com/topics/medicine-and-dentistry/life) (Hornik et al., 2012).

**LOS:** Sepsis caused by pathogens isolated from a sterile site (e.g., blood, urine, cerebrospinal fluid [CSF]) after 3 days of an infant’s life (Hornik et al., 2012; Dong and Speer, 2015).

**Blood sample for culture:** We used standardized culture techniques to reduce false-negative results; a nurse withdrew a 1 mL blood sample by venipuncture prior to initiating antibiotics.

**Tracheal aspirate for culture:** The endotracheal secretions were collected by a respiratory therapist through instilling 1–2 ml of sterile normal saline into the endotracheal tube, which was then re-collected with the assistance of a sterile mucous trap.

**Ventilator-associated pneumonia (VAP)**: Defined as clinical and radiographic changes in intubated infants with onset after 48 h or more of invasive mechanical ventilation, plus isolation of a pathogenic microorganism in the airway aspirate (Cernada et al., 2013).

**Meningitis:** Confirmed by isolation of pathogenic organisms from CSF culture (Geiseler et al., 1980; Brouwer et al., 2010).

**MDROs**: Microorganisms that are resistant to at least one class, of at least one agent, in at least three antimicrobial categories (Magiorakos et al., 2012).

**MDRO-GN:** MDR bacterial infection caused by GN organisms such as *Klebsiella pneumoniae*, *Acinetobacter baumannii*, or *Pseudomonas aeruginosa* (Magiorakos et al., 2012).

**Persistent infection:** Presence of three or more consecutive positive cultures obtained at least 48 h apart during a single sepsis episode (Jen-Fu et al., 2015).

**Concomitant antibiotic:** Any antibiotic administered on the day of exposure to colistin or fluoroquinolone.

**Colistin dosing**: 75,000–150,000 IU/kg/day divided into two to three doses (Nakwan et al., 2019; Lexicomp Online, 2020).

**Fluoroquinolones:** Either ciprofloxacin dosing of 20 mg/kg/day divided into two doses or levofloxacin dosing of 20 mg/kg/day divided into two doses (Kaguelidou et al., 2011; Newby et al., 2017; Lexicomp Online, 2020).

**Antibiotic selection:** In our NICU, the appropriate selection of antibiotics for the treatment of proven sepsis (initiation, duration, and discontinuation) depends on an interprofessional team approach involving infectious disease experts, clinical pharmacists, and neonatologists, taking into consideration the type and duration of previous antibiotics, source of infection, antimicrobial sensitivity results, presence of GN/MDRO outbreak, persistent infection, hemodynamic stability of patients, status of laboratory tests before initiations, pharmacokinetics properties, and availability of medications in the pharmacy.

**Cultures and laboratory follow-up:** Follow-up cultures from blood, tracheal aspirates, CSF, and other suspected sites of infection were obtained after 48–72 h from the initiation of therapy, and when clinically indicated. For evaluation of the possible adverse effects of colistin or fluoroquinolone, creatinine, urea, alanine transaminase (ALT), aspartate transaminase (AST), direct bilirubin, as well as electrolytes including sodium, potassium, magnesium, calcium, and phosphorus levels were collected at the beginning of the treatment, during the treatment (2–3 times weekly or daily, if clinically indicated), and at the end of the treatment.
